# A Proposed Framework for Patient-Focused Policy at the U.S. Food and Drug Administration

**DOI:** 10.3390/biomedicines7030064

**Published:** 2019-08-27

**Authors:** Carrie M. Kuehn

**Affiliations:** College of Professional Studies, Northeastern University, Boston, MA 02101, USA; c.kuehn@northeastern.edu; Tel.: +425-241-6510

**Keywords:** FDA, patient-focused policy, patient preference, patient-focused drug development, guidance

## Abstract

Medical product sponsors are encouraged to include the patient perspective in their medical product development strategy to inform product design, augment regulatory submissions, argue for alternative clinical trial designs, or to support indications in specific patient populations. The goal is to create a patient-focused ecosystem that enables industry to integrate the patient voice throughout the medical product lifecycle. To this end, the U. S. Food and Drug Administration (FDA) has published several guidance documents to provide industry with the expectations and opportunities for conducting patient-focused activities. From an industry perspective, the Center for Devices and Radiologic Health (CDRH) and the Center for Drug Evaluation and Research (CDER)/Center for Biologics Evaluation and Research (CBER) patient-focused policies are complementary. The basic tenets promoted in all FDA patient-focused guidance could apply across therapeutic areas. However, there remain differences in these guidance documents across FDA centers, and there is no framework in place to provide industry with consistent recommendations. Without a coordinated patient-focused policy from the FDA, there is the potential for confusion and a lack of consistency among industry and regulatory decision-makers. The objective of this paper was to propose an alternative framework for patient-focused policy at the FDA, which recognizes the potential for different types of patient input to be used across therapeutic areas and medical product types. Further, these policies need to provide greater clarity on how patient input data is used, so that sponsors may navigate the opportunities to use patient input regardless of the FDA center under which their product is regulated. Creating consistent, coherent, and transparent FDA patient-focused policy will encourage sponsors to obtain patient input more often and with greater certainty of the value that these data may have to their medical product strategies.

## 1. Introduction

Including the patient voice in medical product development and commercialization has never been a greater priority than it is right now. In the United States, legislation aimed at providing the U. S. Food and Drug Administration (FDA) with authority to determine how and when patient data is included in regulatory decision-making has prompted a prolific generation of new guidance and patient-focused policy from the Agency. After the Food and Drug Administration Safety and Innovation Act (FDASIA) was signed into law in 2012, there have been seven draft or final guidance documents from across FDA centers with some focus on including patient perspectives [1,2,3,4,5,6,7]; and three more guidance documents are expected in the next year or two [8]. The proliferation of patient-focused FDA policy and guidance should be good news for patients, patient advocates, and caregivers. However, there remain several challenges that must be addressed before true patient-focused medical product development can be realized.

The objective of this paper is to propose an alternative framework for patient-focused policy at the FDA that recognizes the potential for different types of patient input to be used across therapeutic areas and medical product types. It offers an assessment of the current state of FDA patient-focused regulatory policy, a look at the impact of this policy on the medical products industry, and recommendations for how a coherent patient-focused policy framework can be used to realize the full potential of including the patient voice throughout the medical product lifecycle.

### 1.1. A Brief History of Patient Engagement at the FDA

Patient engagement with FDA has roots in the HIV/AIDS movement of the 1980′s [9,10]. During the AIDS crisis in the 1980s, advocacy organizations such as ACT UP and Project Inform directly engaged the FDA to increase access to life-saving therapies. HIV/AIDS advocates challenged the notion that patients should not have a voice in whether they are exposed to certain risks [9]. Activists also became heavily involved in the conduct of clinical trials, including the use of community-based trials, which were easier to implement, less expensive, and ensured that doctors familiar with HIV/AIDS patients were involved in their treatment and care. One of the most important accomplishments of the HIV/AIDS activist movement was its intimate engagement with the FDA and subsequent influence on regulatory policy. Members of ACT UP and Project Inform came to the FDA prepared with the knowledge necessary to engage meaningfully with FDA experts to identify opportunities for change and to propose solutions [10]. The partnership between HIV/AIDS activists and the FDA led to regulatory and legislative changes that benefited not only HIV/AIDS patients but millions of other patients awaiting life-saving therapies.

The nature of patient engagement at the FDA has changed a lot over the last 30 years. In the 1990′s, the Patient Representative Program provided an opportunity for patients, patients’ families, and caregivers to provide the patient perspective as participants on FDA Advisory Committees [11,12]. Patients and patient advocacy groups have also participated in open public hearings, public policy, and town hall meetings, and have provided written comments to advisory committees and in response to proposed FDA rules in the Federal Register. The opportunity for patient engagement with the FDA increased dramatically in 2012, with the enactment of the Food and Drug Administration Safety and Innovation Act (FDASIA) [13]. For the first time, the FDA was mandated by law to: *develop and implement strategies to solicit the views of patients during the medical product development process and consider the perspectives of patients during regulatory discussions* (FDASIA Sec 1137). Under the Medical Device User Fee Act (MDUFA) III reauthorization in 2012, the FDA’s Center for Devices and Radiologic Health (CDRH) committed to meet with patient groups to better understand and characterize patient perspectives [14]. Similarly, under the Prescription Drug User Fee Act (PDUFA) V reauthorization, the Center for Drug Evaluation and Research (CDER) and Center for Biologics Evaluation and Research (CBER) launched the Patient-Focused Drug Development (PFDD) program aimed at obtaining patient perspectives on specific diseases and their treatment. Opportunities for patient involvement at the FDA continued to evolve, including establishment of the Patient Engagement Advisory Committee (PEAC), members from which provide input to the CDRH on issues, such as guidance and policies, clinical trial design, patient preference design, device labeling, and other patient-related topics [15].

The FDA’s patient-focused efforts dramatically expanded following enactment of MDUFA IV and PDUVA VI as part of the Food and Drug Administration Reauthorization Act (FDARA) in 2017 [16]. New programs were implemented across FDA centers, including the development of FDA resources and expertise focused on patient preference information (PPI), the assessment of approaches to including PPI and patient-reported outcome (PRO) and clinical outcome assessment (COA) data in regulatory submissions and clinical trial study design, and the identification of therapeutic areas with preference-sensitive questions that could be answered using PPI data [17,18]. Other activities include establishment of Patient Affairs staff to provide a central point for patient engagement activities at the Agency, and the creation of a memorandum of understanding (MOU) between the FDA and the National Organization of Rare Disorders (NORD) to identify the urgent needs of patients with rare diseases and incorporate patient experience into drug development programs [19].

### 1.2. FDA Patient-Focused Policy and Guidance

**Figure 1:**
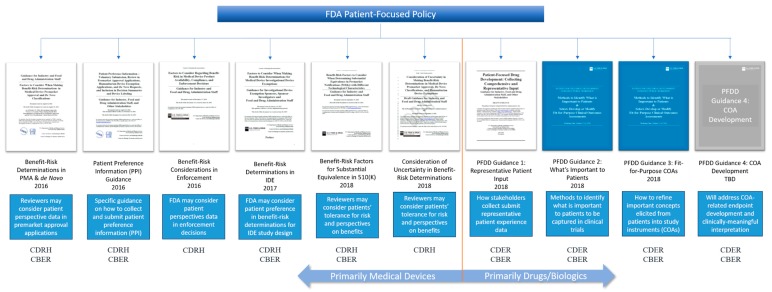
U. S. Food and Drug Administration (FDA) guidance on the use of patient preference or experience data in regulatory decision-making.

Other patient-focused activities at the FDA include the ongoing work of the FDA Patient Council (originally organized as the FDASIA 1137 working group) to coordinate patient-focused policy efforts across centers. The FDA Patient Council was established in 2016 to “*coordinate and integrate the role of patient perspectives in regulatory decision-making over the total product lifecycle* [20].” At a recent Patients as Partners conference, a panel of FDA staff shared their efforts to work synergistically across review centers to coordinate patient engagement efforts [21]. My interactions with FDA staff working on patient-focused policy initiatives echo the sentiments of the panel. There is no doubt that patient input is valuable across therapeutic areas and regulatory centers. However, when it comes to understanding just what the FDA expects from the medical products industry regarding patient input, there is still confusion. To illustrate this point, it is helpful to look at the variety of patient-focused guidance currently available from FDA (Figure 1).

### 1.3. FDA Patient-Focused Policy

Since 2016, eight final or draft FDA guidance documents have been published with some mention of the use of patient input. Additionally, there are two guidance discussion documents and one more planned guidance with discussion still to be scheduled. The guidance documents can be broken down into three focus areas: patient preference, benefit-risk determination, and patient experience. Examining these three areas reveals why industry is so unsure of how to interpret the FDA’s thinking about the use of patient input in regulatory submissions and beyond.

### 1.4. Patient Preference

Patient preference has been primarily within the purview of the CDRH since dissemination of the Patient Preference Information (PPI) guidance in 2016 [3]. The PPI guidance is intended to provide industry with some instructions on what constitutes patient preference data and how it can be used. However, the guidance falls short of explaining exactly how to go about obtaining relevant patient preference data, how to ensure it is clinically meaningful, or precisely what type of data will be acceptable to the CDRH in any given regulatory context. The Medical Device Innovation Consortium (MDIC) has worked with the FDA to fill some of these gaps by publishing a summary of methods for use in patient-centered benefit-risk studies (a term often used interchangeably with patient preference study), affectionately referred to as the Compendium [22]. While valuable, the MDIC Compendium does not address the FDA’s expectations for industry or how to design a patient preference study to fit the regulatory needs of the sponsor. The result is that FDA has received only a dozen or so submissions of patient preference studies since publication of the guidance in 2016. Of these, only two patient preference studies have substantially impacted regulatory decision-making [23,24]. Further, except for the FDA’s original obesity study, none of the submitted studies have provided an example of best practices that the industry could use as a benchmark. The lack of best practice data has limited transparency into the types of studies the FDA will accept and for what purpose, and kept device sponsors somewhat cautious about the use of patient preference in their regulatory strategy.

### 1.5. Benefit-Risk Determinations

The benefit-risk guidance documents—all five of them—from the CDRH (sometimes along with CBER) cover benefit-risk determinations in all forms of medical device pre-market pathways (510(k), *de Novo,* and PMA) and clinical trials (IDE) [1,2,4,5,7]. It is important to note that all FDA guidance documents that mention the use of patient preference data in benefit-risk determinations are focused on medical devices, not drugs or biologics. It would be understandable if the medical products industry interpreted this to mean that FDA will only consider patient preference data in medical device benefit-risk determinations. Furthering this interpretation is the FDA’s current five-year plan to enhance benefit-risk determinations in drug and biologics decision-making [25]. Based on the emphasis of patient preference in CDRH benefit-risk guidance documents, we should expect that CDER and CBER would consider patient preference as well, but they do not. Instead, CDER and CBER focus on the use of patient experience data, further separating the use of different types of patient input across medical product lines.

### 1.6. Patient Experience

Patient experience has been the primary focus of CDER and CBER. After establishing the PFDD program, CDER-CBER launched into the implementation of PFDD meetings to capture the patient experience in a variety of disease areas. These meetings continue today as externally-led PFDD, and now Patient-Focused Medical Product Development (PFMPD) meetings [26]. Following enactment of the 21st Century Cures Act and FDARA, CDER-CBER were required to generate guidance documents that provide specific steps for industry to follow to include patient experience data in regulatory submissions [16,27]. These four guidance documents are focused on two things: patient experience and the development of clinical outcome assessments (COAs) by which patient experience can be systematically captured using validated instruments in clinical trials. Given that these concepts are being primarily dealt with by CDER and CBER, the medical product industry could be forgiven for thinking that patient experience is only relevant to drug and biologics development (see benefit-risk determinations above). This interpretation, however, fails to consider the fact that patient experience is relevant to all medical products—devices, drugs, biologics, and combination products. Separating the type of patient input by medical product limits the use of patient data by sponsors, and it segregates resources and expertise at the FDA by review center. Instead, patient input of all types, including patient preference, patient risk tolerance, and patient experience should be considered across all FDA review divisions regardless of the medical product. A cohesive policy framework that organizes patient-focused policies such that they can be applied to all medical product reviews is warranted.

### 1.7. Impact of Current Patient-Focused Policy on Industry

The FDA is interested in capturing a variety of patient input across medical products. However, despite the efforts of the FDA Patient Council, and reassurances that FDA review centers are coordinating the process, the current system of patient-focused policy at FDA is segregated by medical product type. This segregation gives the false impression that only certain types of patient input are appropriate for certain types of medical products: patient preference for devices and patient experience for drugs and biologics. However, this impression does not reflect reality. There must be a better way to communicate the FDA’s expectations for submission of patient input data, such that industry feels empowered and encouraged to collect all types of patient input regardless of the type of medical product they are developing.

**Figure 2:**
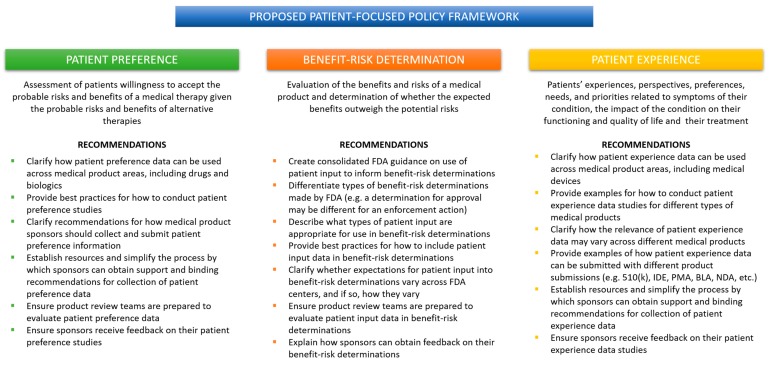
Proposed policy framework for FDA patient-focused policy.

When a medical product sponsor wants to know what the FDA is thinking about a topic, it looks to FDA guidance. More specifically, a sponsor will look at guidance from their relevant review center. That means a medical device company that wants to include the patient voice in their regulatory submission will turn to the CDRH guidance, which will inevitably focus on patient preference. The same could be said for a drug or biologics company that will rely primarily on guidance from CDER and CBER to gain insight into the FDA’s thinking about what kind of patient data is appropriate. That drug or biologic company will find several CDER/CBER guidance documents focused on patient experience, but none that mention patient preference. Unfortunately, this means that valuable patient experience data may never make its way into a medical device regulatory submission or anywhere in the device lifecycle. Similarly, patient preference data, while relevant to the development of drugs and biologics, may never be captured by a drug or biologics company because of the CDER-CBER focus on patient experience and COAs. Without clear, cohesive guidance from the FDA, industry will either opt-out of including patient input or fail to capture the most relevant data for their product. What is needed is a patient-focused policy framework organized around the type of patient data rather than the type of medical product (Figure 2).

### 1.8. A Proposed Framework for FDA Patient-Focused Policy

A coordinated patient-focused policy framework from the FDA would not be complicated. Current and proposed FDA guidance contains within them all the building blocks for a coordinated policy framework. As outlined in Figure 2, three specific guidance documents, co-sponsored by the three main review centers: CDRH, CDER, and CBER, would go a long way to providing industry with greater clarity and predictability about how, when, and which patient input data can be used in a regulatory submission and within the development cycle.

For example, the current PPI guidance could be revised to expand its applicability to all medical products. The principles of conducting patient preference studies will be similar across therapeutic areas, and expectations for rigorous methodology and statistical analysis are medical product agnostic. CDRH already has experience training device review teams to consider patient preference data. Surely, this experience could be used to implement similar training across CDER/CBER review teams. It makes sense to have device-, drug-, and biologic-specific provisions in the guidance when appropriate, but the overall guidance would provide a cohesive picture of the FDA’s expectations for patient preference data in regulatory decision-making across the Agency. When it comes to patient preference, the FDA could leverage the work they have already done to build the PPI program and to expand it to encompass more than medical devices.

Addressing the need for consolidated benefit-risk determination guidance could be more challenging. There are so many guidance documents focused on different types of regulatory submissions and considerations; it is unclear how best to approach the problem. One option would be to carve out a single guidance document specific to how patient input can inform benefit-risk determinations of various kinds. The FDA could take the bits and pieces related to the use of patient input in benefit-risk determinations from existing and proposed guidance documents to create one cohesive policy that covers all medical products across the Agency. While ambitious, it is not impossible, and it would provide industry with one guidance from which they can find best practices for developing their patient-focused benefit-risk product profiles.

Like patient preference, the FDA could generate a single guidance focused on the use of patient experience data in regulatory decision-making. The current PFDD guidance is still useful; it provides important information about how to obtain representative patient experience data. Why not expand its purview to the collection of patient experience data for medical device products? Again, the methods and expectations for rigor should not differ across review centers. The FDA could provide examples of the use of patient experience data in the review of a variety medical products and illustrate how the different review centers would consider these data as part of their regulatory determinations. So much work has already been done, patient experience is another area in which the FDA can leverage its experience and knowledge to communicate more clearly to industry just how these data can be used to support product reviews.

For the proposed framework to be successful, these guidance documents regarding patient preference, benefit-risk tolerance, and patient experience must be broadly applicable to all medical products, recognizable by the industry as pertaining to multiple product types and therapeutic areas, and contain provisions explaining how patient input data will be used in each context. Further, each guidance should be internally consistent with the others, contain cross-referenced content where applicable, and provide accessible resources for sponsors. Finally, the FDA must ensure that review teams are familiar with all forms of patient input data and be prepared to consider them as part of the regulatory review process. Transparency from the FDA on how and when patient input data is used will encourage greater participation from industry in these patient-focused initiatives.

## 2. Conclusions

Patient-focused policy at the FDA is a good idea that needs to be operationalized in a more coordinated and industry-supportive way. If current fiscal plans do not allow for the revision or development of the proposed guidance, the FDA has many vehicles available to disseminate a coordinated approach to these issues, including their newly updated website. The FDA needs to provide assurance and methodological support to the medical products industry if they want to see more patient input data being used during medical product development and submitted during regulatory processes. For its part, the medical products industry needs to advocate for transparency and accountability from the FDA to justify the costs of investing in patient input studies. While the focus in this paper has been on the use of patient input in regulatory submissions, patient-focused guidance put forth by the FDA should make it clear how patient input can be relevant throughout the total product lifecycle of a product. To the extent that FDA patient-focused policy can be organized and clarified using a simplified policy framework, it will help ensure that patient input is effectively solicited at all possible points along the medical product total product lifecycle.
